# Opportunities for Early Cancer Detection: The Rise of ctDNA Methylation-Based Pan-Cancer Screening Technologies

**DOI:** 10.3390/epigenomes6010006

**Published:** 2022-02-04

**Authors:** Nicolas Constantin, Abu Ali Ibn Sina, Darren Korbie, Matt Trau

**Affiliations:** 1 Centre for Personalised Nanomedicine, Australian Institute for Bioengineering and Nanotechnology (AIBN), Corner of College and Cooper Roads (Bldg 75), The University of Queensland, Brisbane, QLD 4072, Australia; n.constantin@uq.edu.au (N.C.); a.sina@uq.edu.au (A.A.I.S.); 2 School of Chemistry and Molecular Biosciences, The University of Queensland, Brisbane, QLD 4072, Australia

**Keywords:** cancer screening, liquid biopsy testing, circulating tumour DNA, cancer epigenetics, DNA methylation biomarkers, tissue-of-origin prediction, multi-cancer early detection, combinatorial analysis, positive predictive value

## Abstract

The efficiency of conventional screening programs to identify early-stage malignancies can be limited by the low number of cancers recommended for screening as well as the high cumulative false-positive rate, and associated iatrogenic burden, resulting from repeated multimodal testing. The opportunity to use minimally invasive liquid biopsy testing to screen asymptomatic individuals at-risk for multiple cancers simultaneously could benefit from the aggregated diseases prevalence and a fixed specificity. Increasing both latter parameters is paramount to mediate high positive predictive value—a useful metric to evaluate a screening test accuracy and its potential harm-benefit. Thus, the use of a single test for multi-cancer early detection (stMCED) has emerged as an appealing strategy for increasing early cancer detection rate efficiency and benefit population health. A recent flurry of these stMCED technologies have been reported for clinical potential; however, their development is facing unique challenges to effectively improve clinical cost–benefit. One promising avenue is the analysis of circulating tumour DNA (ctDNA) for detecting DNA methylation biomarker fingerprints of malignancies—a hallmark of disease aetiology and progression holding the potential to be tissue- and cancer-type specific. Utilizing panels of epigenetic biomarkers could potentially help to detect earlier stages of malignancies as well as identify a tumour of origin from blood testing, useful information for follow-up clinical decision making and subsequent patient care improvement. Overall, this review collates the latest and most promising stMCED methodologies, summarizes their clinical performances, and discusses the specific requirements multi-cancer tests should meet to be successfully implemented into screening guidelines.

## 1. Introduction

Detecting cancer at an early stage (i.e., stages I and II), when primary tumours are still localized and have not spread to the surrounding tissues, remains the best strategy for successful treatment and increased overall survival rate [1,2,3,4]. This is because, when detected early, tumours can be effectively treated by surgical resection, localized radiotherapy or chemotherapy; in contrast, overall survival rates decline drastically with late-stage diagnosis, particularly in metastatic cases which are frequently associated with poor prognosis. Early detection also enables reduced medical costs as expensive and long-term treatment accompanying later disease detection are usually no longer needed. However, conventional diagnosis of cancer burden still relies on reporting of symptoms and medical imaging of a single tissue at a time, followed by tissue biopsy for histopathological analysis when a mass is detected. Such a process is often ineffective for early cancer detection as it relies on symptomatic and phenotypic changes that typically appear at an advanced stage of malignancy [5]. However, new methodologies for sensitive and accurate detection of multiple cancer types simultaneously are emerging as novel strategies for improving early cancer detection rate with the potential to also benefit population health and economics [6].

One of the most promising avenues to early-stage disease detection is liquid biopsy testing from blood, and to a lesser extent, urine, as these body fluids are known to carry specific cancer biomolecules (e.g., circulating free DNA, RNA, exosomes and circulating tumour cells) that directly originate from malignant tissues. These biomarkers are released by cancer cells through various mechanisms and have been reported to harbour specific signatures that could reflect tumour burden, stage, or location, a precious source of clinical information for cancer diagnosis as well as treatment monitoring [7,8,9,10,11]. The analysis of circulating tumour DNA (ctDNA) has been of particular interest over recent years because of its remarkable stability in body fluids and its early occurrence during disease development.

Two different strategies are currently available for interrogating ctDNA from blood in biomarker discovery and cancer diagnostic development. The most established method consists of using next-generation sequencing analysis to probe for specific genetic abnormalities (e.g., somatic mutations) associated with cancer which quantitative genotypic information of tumour burden provides based on variant allele frequency (VAF) [12,13,14], as well as identifying potential therapeutic options (e.g., mutations related to BRAF and PARP inhibitor therapy). Although the recurrence of certain DNA mutants gradually increases with tumour progression, their presence in the complex blood matrix during the onset of malignancies may be so limited (VAF << 1%) that the analytical sensitivity of current methods is still challenged for their accurate detection in early disease settings. A promising alternative method for interrogating trace amounts of ctDNA involves screening for changes in DNA methylation at CpG dinucleotides—a global epigenetic marker involved in gene activity regulation. DNA methylation reprogramming have been reported to be a hallmark of disease aetiology and progression and could offer additional opportunities for the development of biomarker fingerprint for early cancer detection [15,16,17,18].

**Figure 1:**
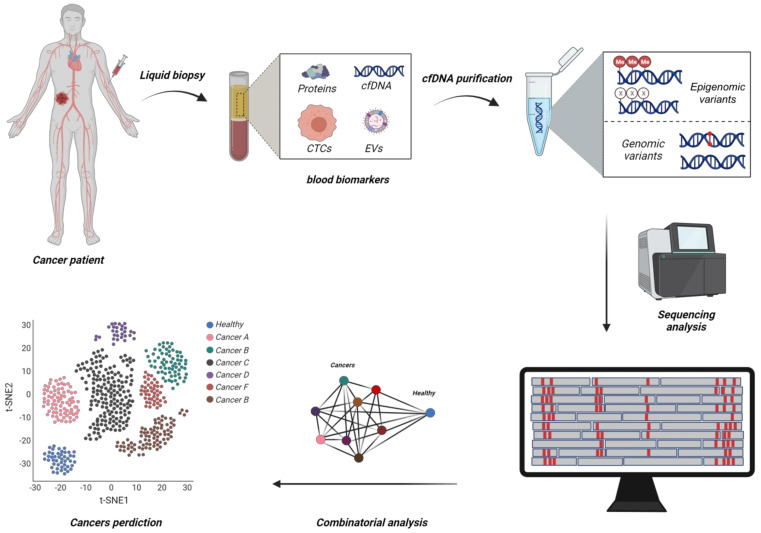
Schematic representation of emerging diagnostic methodologies using combinatorial analysis of large circulating tumour DNA (ctDNA) biomarker panels to develop single test-based Multi Cancer Early Detection (stMCED) blood-based test. Circulating tumour cells (CTCs), Extracellular vehicles (EVs), CpG Methylation (Me) (Created with BioRender.com).

With the recent advances in molecular technologies, the last decade has encountered a true revolution in genomic and epigenetic profiling methods and an exhaustive number of these informative regions have been reported for clinical utility [15,16,17,18]. The use of bioinformatics to analyse these large panels of biomarkers and build cancer-specific classifiers has been at the forefront of the research space to achieve high cancer detection power (Figure 1) [19]. In this technological race, a recent flurry of new methodologies has emerged, each one reporting different diagnostic performances for early disease detection and claiming potential clinical benefits. However, such performances are mostly reported in terms of the usual sensitivity and specificity parameters without mentioning the associated positive predictive value (PPV), a metric that may better reflect diagnostic accuracy in our opinion. Herein, we first articulate the challenges associated with the current diagnostic paradigm to efficiently identify early-stage of malignancies and discuss the technological parameters surrounding clinical risk–benefit required to achieve potential translation. We then review the latest advances in cancer epigenetics for the development of single tests for multiple cancer early detection (stMCED), having the potential to increase overall cancer detection rate and improve patient survival outcome, and provide their extrapolated PPV when possible (see methodology Section 8). Together, this review showcases the unique potential of DNA-methylation biomarkers to predict tumours of origin from liquid biopsy testing, clinical information highly valuable for patient care but difficult to retrieve from genetic mutation analysis alone.

## 2. Challenges Associated with the Current Screening Paradigm to Efficiently Identify Early-Stage Malignancies

Individuals with early-stage malignancy are usually asymptomatic and remain untested; it is only with the presentation of symptoms that diagnosis occurs, a situation often associated with a more advanced stage of the disease and poor prognosis. Faced with this, an ideal approach for increasing early detection rate would appear to be a clinical recommendation to perform a general cancer test on an annual basis for all asymptomatic individuals in the general population. However, to be implemented for general screening of healthy individuals, a test would presumably need to achieve a maximal specificity of 100% to limit the false positive rate to 0% and ensure adequate clinical risk–benefit. Indeed, every false-positive result would misguide any patients towards further clinical testing, with concomitant negative impacts on patient mental health and healthcare system burden. For instance, if 1 billion tests were to be performed using a diagnostic test with a specificity as high as 99.9%, it would still result in 1 million individuals incorrectly classified as having disease, all of whom would have to endure an unnecessary and expensive diagnostic odyssey. Given this false-positive problem, screening the general population annually seems unrealistic and may not be a practical strategy to improve early cancer detection rate.

As an alternative, conventional guidelines recommend cancer screening for at-risk individuals (e.g., elderly, smokers, with an existing genetic disorder, or with a family history of cancer) mostly targeting age-restricted populations [20]. For instance, women aged 45 years should start undergoing annual screening mammography for breast cancer detection, with the specificity of modern screening digital mammography being 88.9% [21]. Men from 50 years of age should undergo prostate cancer screening using prostate-specific antigen testing which has been reported to have a specificity of 91% [22]. Adults aged 45 years should start regular colorectal cancer screening with various options available, including minimally invasive stool-based assays, and with optical colonoscopy for structural examination of the bowel remaining the gold standard recommendation (any positive results from non-colonoscopy screening tests should be followed up by a timely colonoscopy) [23]. Different performance for coloscopy screening has been reported in the literature, but a meta-analysis has estimated that the specificity to detect adenomas of >6 mm and >10 mm in size was 94.2% and 88.7%, respectively [24]. Women from 21 to 65 years old should be screened for cervical cancer regularly with the option of using cytology testing (conventional Pap test) alone on a 3-year basis, with an estimated specificity of 92% [25,26]. Current and former smokers (with a 30 pack-a-year smoking history) aged 55 to 74 years should undergo regular lung cancer screening, with a low-dose computed tomography (CT) scan reported to have a specificity of 87.2% [27].

As detailed above, the current screening paradigm relies on methods that have been developed to interrogate one specific organ at a time, while individuals may undergo screening for more than a single disease. The major drawback of such multimodal screening programs is that the cumulative risk for an individual to obtain a false positive result will inevitably increase with the number of screening tests performed. *Croswell* et al. conducted a randomised controlled trial to evaluate the incidence of false-positive results in repeated multimodal cancer screening [28]. The study involved 68,436 participants aged 55 to 74 years and investigated screening modalities, which were advocated at the time of the trial, for prostate (prostate-specific antigen and digital rectal examination), lung (chest radiography), colorectal (flexible sigmoidoscopy), and ovarian cancer (cancer-antigen 125 and transvaginal ultrasonography). Over a period of three years, the cumulative risk of false-positive findings after four examinations was 36.7% for men and 26.2% for women; and after 14 examinations was 60.4% and 48.8% for men and women, respectively. Given the magnitude of cumulative false positives, along with potential invasiveness and related cost, it is clear that the iatrogenic burden resulting from multiphasic screening programs would represent a non-negligible risk to the individual undergoing testing.

In addition, a conceptual limitation of the current guidelines to effectively increase early cancer detection rate is that screening is only recommended for a handful of cancer [20]. This strategy only covers a fraction of diseases that an individual will potentially develop in his life span and thus limit the possibility to detect the full spectrum of prevalent malignancies in the asymptomatic population.

Moreover, some of the conventional screening methods such as optical colonoscopy or cervical cytology testing can involve invasive procedures that could cause discomfort and, in some cases, complications [29,30]. Other methodologies exposing patient’s body to radiation such as CT scan or mammography could also come with potential risk when undergoing repeated testing [31,32]. The emergence of minimally invasive methodologies such as blood, urine, or stool-based testing are of promising avenue to reduce invasiveness and risk, as well as facilitate uptake and repeated testing in screening settings. However, one recurrent challenge for detecting early-stage of malignancies from liquid biopsy testing is the technical sensitivity that methodologies should achieve to correctly identify tumour specific molecules and associated signatures that are only present in a trace amount in body fluids [33].

**Figure 2:**
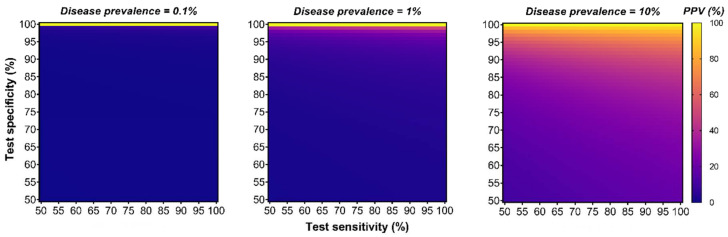
Although many diagnostic tests focus on optimizing sensitivity, specificity, and disease prevalence have the strongest impact on the Positive Predictive Value (PPV). This series of heatmap graphs illustrate the relationship of a diagnostic test PPV (%) in a function of different sensitivity and specificity parameters (both ranging from 50 to 100%) for a hypothetical disease prevalence fixed to 0.1% (**left**), 1% (**middle**) or 10% (**right panel**). (See the methodology Section 8 for the PPV equation used).

The technological performances of diagnostic assays are generally reported in terms of specificity (i.e., the true negative rate) and sensitivity (i.e., the true positive rate) as paired indicators. Integrating both parameters into a single indicator of diagnostic performance could help compare the effectiveness of competing tests and facilitate decision making. For instance, the Diagnostic Odds Ratio (DOR) can be utilized as such a single indicator and is defined as the odds of disease in positive testing over the odds of disease in negative testing [34]. However, the DOR is independent of the disease prevalence (i.e., the total number of people with the disease in a specific population at a given time) which is also an important parameter affecting the detection rate performance of a screening test [35]. For instance, screening the women population for breast cancer will yield a significantly higher detection rate compared to screening the men population as the prevalence of breast cancer is higher for women. One single indicator of diagnostic performance also integrating the disease prevalence is the Positive Predictive Value (PPV); a measurement corresponding to the likelihood that a patient returning with a positive test will truly have the disease when screening a specific population. This measurement takes into account the three aforementioned parameters (specificity, sensitivity and disease prevalence) and may better reflect the global performance of a test for predicting detection rate accuracy in screening settings. Although the PPV is influenced by the disease prevalence and cannot be generalized between populations, it can provide a useful metric to evaluate potential harm–benefit resulting from testing. Herein, we have modelled the effect of these parameters on the PPV (see methodology section for detailed formula) and show that maintaining a high specificity and increasing the disease prevalence is paramount to mediate stronger PPV, as opposed to increasing sensitivity, which had a minimal influence (Figure 2).

In summary, the limited number of cancer types targeted by guidelines-recommended screening as well as conventional multimodal testing programs accepting relatively low specificity are the major limitations to efficiently improving early cancer detection rate. One promising strategy to potentially address these limitations and yield higher PPV is implementing a high specificity blood-based test for screening multiple cancer types simultaneously.

## 3. The Clinical Potential of Implementing a Single Test for Multiple Cancer Early Detection (stMCED)

While individuals may be identified as at-risk for a particular cancer type (e.g., lung cancer), their combined risk of developing any other cancer (e.g., prostate, colorectal, melanoma, head and neck, etc.) is substantially greater than the individual risk of cancer they are being tested for [3,4]. It is this area—testing at-risk individuals and the ageing population for the presence of multiple different cancers—that stMCED testing from blood is likely to achieve the most clinical benefit. Using a single blood-based test to target multiple cancers simultaneously would first benefit from (i) a fixed specificity and thus a fixed false-positive rate. This would allow limiting the number of false-positive findings as opposed to the cumulative false-positive rates resulting from conventional multimodal screening programs. Therefore, it could help improve patient management and mental health as it would reduce the number of unnecessary confirmation tests and associated iatrogenic burden. (ii) Screening individuals for multiple cancer types simultaneously would also allow covering a broader spectrum of prevalent malignancies and benefit the test from their aggregated prevalence as opposed to traditional mono-cancer tests. This could potentially increase the chance to detect any malignancies at an earlier stage and help reduce cancer-specific mortality in age-restricted populations’ screening settings [36,37]. Together, increased specificity and aggregated diseases prevalence could boost the positive predictive value (PPV) of stMCED (Figure 2), a strategy with the potential to improve early cancer detection rate efficiency with a better harm–benefit tradeoff [38] as opposed to conventional multimodal screening settings.

## 4. The Potential Value of Methylated-cfDNA for Developing stMCED

Although cancer is mainly considered to be a disease driven by genetic mutations, epigenetic alterations are also a critical factor for the onset of malignant diseases and tumours development. DNA methylation at CpG dinucleotides—enzymatically mediated by DNA Methyltransferase—is a global epigenetic mark used to regulate gene expression and signalling pathways activity to control diverse cellular functions. The subsequent regions of the genome that are demarked for transcriptional activation or silencing are of critical importance to define the functionality of a cell and maintain proper homeostasis [39]. As such, DNA methylation is known to play a critical role in various biological processes such as embryonic development, cellular pluripotency, differentiation, and cellular ageing [40,41], and aberrant methylation patterns are now associated with a growing number of diseases [42]. In particular, neoplastic cells can manipulate epigenetic mechanisms by remodelling and overriding normal DNA methylation patterns thereby triggering activation of oncogenic pathways for malignant transformation. Of note, these aberrant DNA methylations are associated with diverse genomic classes such as transposable elements or sequences enriched with CpGs dinucleotides, and such global epigenetic changes can serve as an alternative to somatic mutation to fuel cells with oncogenic properties and represents a hallmark of disease aetiology and progression [43,44,45,46].

During cancer-related methylome reprogramming, two major epigenetic abnormalities occur across the genome—DNA hypermethylation and hypomethylation. DNA hypermethylation tends to cluster at regulatory regions enriched with CpG islands which are normally unmethylated and function to control the activity of anticancer mechanisms but become silenced upon hypermethylation. Notably, the ability to modulate the functional state of such promoter regions acts as an alternative to somatic mutations to block the activity of tumour suppressors and DNA repair genes, thereby promoting cancer progression [47,48,49]. Conversely, cytosines located in non-CpG islands are highly methylated in healthy cells but experience a general loss of methyl groups during neoplastic transformation [18,50]. Although hypomethylation mechanisms remain less understood, some evidence suggests that it could trigger aberrant activation of cancer-germline genes to promote key oncogenesis processes and tumour development. Together, these bimodal epigenetic features have been reported to be a common mechanism of most cancer types producing specific methylation patterns that significantly differ from normal epigenomes, and have therefore been proposed as informative surrogates for somatic mutations biomarkers typically associated with cancer [47,49,50,51]. In particular, there are two key aspects of cancer epigenetics that make DNA methylation biomarkers valuable candidates for developing stMCED: (i) their availability and remarkable stability in most body fluids as part of circulating-free DNA (cfDNA) which enable minimally invasive liquid biopsy testing, and (ii) their early appearance during malignant transformation is a promising avenue for early disease detection, a key strategy for increasing chance of curative treatments and reducing cancer-related mortality.

The potential outlined above of clinical epigenetics for early disease detection has resulted in a flurry of in-vitro diagnostic (IVD) tests based on DNA methylation analysis of ctDNA, with the majority of these tests targeting panels of hypermethylated CpGs associated with specific oncogenic genes, typically for the detection of a single specific cancer type [52,53,54]. For example, the UroMark test is one promising assay to detect bladder cancer (BC) from residual DNA in voided urine. This test targets 150 CpGs loci using next-generation bisulfite sequencing and was able to classify primary BC from non-BC urine samples with 98% sensitivity and 98% specificity [55]. The EpiproLung test can be used as an alternative to low dose computed tomography to screen lung cancer among the high-risk population. This PCR assay interrogates the methylation status of SOX2 and PTGER4 genes from plasma samples and was able to discriminate lung cancer from normal subjects with 67% sensitivity at a fixed specificity of 90% [56]. EarlyTect assay can detect early stages of colorectal cancer (CRC) using quantitative PCR methods to analyse DNA methylation level of SDC2 gene from stool samples. This test has recently reported an overall sensitivity of over 90% and a specificity of the same value to detect CRC regardless of stages [57]. The HCCBloodTest has been developed to detect hepatocellular carcinoma using a PCR-based assay to analyse hypermethylation of the SEPT9 promoter from blood-derived cfDNA with 91% sensitivity and 87% specificity [58].

Although these ctDNA methylation-based IVD assays relying on minimally invasive analysis have significantly improved the standard of care methods and highlight the potential of cancer epigenetics, they are still designed to target one specific type of cancer and thus do not fully address the aforementioned limitations. One potential avenue is to exploit artificial intelligence methods to analyse large datasets and build more comprehensive DNA methylation panels to predict specific cancer types, and eventually combining them for the development of a single test for multi-cancer early detection. However, one challenge associated with stMCED is the ability to correctly identify the tumour of origin after a blood-test returns positive. With that regard, acquired DNA methylation state can be tissue-specific and maintained during neoplastic transformation, creating epiclone signatures that could allow tumour of origin identification [44,59]. This is where DNA methylation biomarkers could show additional potential as this is key information to guide follow-up clinical decision making and facilitate subsequent patient care and management.

## 5. Criteria for Developing Efficient stMCED

Methodologies for sensitive and accurate detection of multiple cancer types using a single assay are emerging as a promising paradigm for potentially increasing early cancer detection rate and improved harm-benefit tradeoff when screening asymptomatic individuals in the ageing and at-risk populations. To be successfully implemented into screening programs and effectively improve health impact while minimizing the associated risk, stMCED would need to meet the following specific criteria [60]. (1) The sensitivity of the method must be very high to allow testing from blood and accurately detect minimal levels of ctDNA present at an early stage of malignancies, and ideally limit overdiagnosis of benign neoplasms. (2) The specificity of the test must be very high to minimize the iatrogenic and financial burden of false-positives. (3) The ability to identify the tumour of origin for the true positive patients would be highly valuable to guide subsequent clinical decision making, as there is no prior knowledge of the disease location at an early stage of cancer disease. (4) The test should include a high number of cancer types, preferentially targeting aggressive diseases with a high incidence rate, to take advantage of the overall prevalence and boost the PPV to maximize the chance of any cancer detection. (5) The cost of the test must be minimized to ensure population uptake test and allow the repetitive screening. Together, these criteria should be taken into account and considered in light of each other when developing blood-based stMCED technologies. In particular, efforts should favour increasing the specificity and the aggregated prevalence over the sensitivity as these two parameters have a significantly greater impact on the positive predictive value of a diagnostic test (Figure 2).

## 6. Methodologies for stMCED Screening

The recent advances in sequencing and PCR technologies have certainly allowed the development of powerful molecular assays enabling high throughput and large-scale analysis of the human genome. With the help of bioinformatics, it is now possible to analyse these extensive genomic (or epigenomic) datasets to decipher combinatorial biomarkers highly specific to different cancer types. These computational methodologies have opened new avenues for the discovery and the generation of larger, more comprehensive and more performant biomarkers panels suitable for stMCED. Here, we discuss the most promising blood-based diagnostic assays utilizing ctDNA analysis for the detection and potential identification of multiple cancer types at an early stage of disease and compare the performance of methylation-based and other technologies (see methodology Section 8 for the literature search criteria and Table 1 for an overview of selected technologies).

### 6.1. Non-Methylation Based Assays

#### 6.1.1. DEEPGEN™

DEEPGEN™ assay is a blood-based ctDNA test that has been specifically developed for multi-cancer detection in early disease settings [61]. The authors have used a bioinformatics to identify a broad panel of cancer-specific genomic variants that are present at very low frequency in blood. The assay targets 3062 of these mutant fragments (including single nucleotides polymorphisms, multi nucleotide polymorphism, and short insertion or deletion) using proprietary primers for advanced PCR and next-generation sequencing analysis, resulting in high capture efficiency at VAF down to >0.09% [62]. DEEPGEN™ has been used in an initial comprehensive prospective study for the detection of seven different types of cancers (bladder, prostate, lung, liver, pancreatic, colorectal, and breast) using a cohort of 260 cancer samples across stages I-IV (27% stage I, 21% stage II, 29% stage III, 10% stage IV and 13% undetermined) and 415 healthy controls. Machine learning algorithms have been designed to analyse mutations frequencies data obtained from their genomic target panel to build classifiers deriving prediction scores of cancers. One general classifier of cancer has been trained using all the samples, while separate cancer-specific classifiers have been trained using samples derived from a single organ only. The analytical sensitivity of the full model was 57% at a selected specificity of 95% with a corresponding area under the receiving operating curve (AUROC) of 0.9, while the detection sensitivity increased from 51% in stage I to 67% in stage IV. The performance of the individual cancer classifiers had overall sensitivities that differed between cancer types: being the lowest for breast (30%) and colorectal cancers (42%), and the highest for prostate (72%) and bladder cancer (80%). (Overall analytical performances of DEEPGEN™ are reported in Table 2).

Together, these preliminary results indicate a promising clinical capability of the DEEPGEN™ assay to differentiate healthy individuals from those with a disease for seven different cancer types. Although the reported clinical performance to detect early-stage malignancies has the possibility of improving patient care, higher specificity should be favoured over higher sensitivity to better benefit the platform performance in terms of its PPV. As such, we have estimated that increasing the specificity from 95% to 99% would increase the overall PPV from 29.9% to 61.7% when considering screening the Australian population in the 55–64 age-restricted group (Table 8). In addition, the methodology is still at an initial development stage and the authors have not published pieces of evidence yet about the ability of the platform to accurately predict the tumour of origin of a sample that tested positive. Although larger datasets are needed to improve the classifiers’ performance and independent cohort for further validation of the models, the interrogation of genomic variants only may limit the possibilities of identifying specific cancer given the heterogeneous nature of somatic mutations.

#### 6.1.2. CancerSEEK

CancerSEEK is one promising blood-based test developed to detect and locate the presence of eight different types of cancers (ovary, liver, stomach, pancreas, oesophagus, colorectum, lung, or breast) using an innovative multi analytes approach—targeting both genetic and protein biomarkers [63]. The first component of the assay relies on sequencing of cfDNA targeting 16 different oncogenic driver genes which comprise a total of 1933 distinct genomic positions frequently mutated in cancers. To achieve the high sensitivity required to detect rare mutations associated with early-stage cancer, *Cohen* et al. [63] have designed a robust multiplex PCR assay to amplify the targeted mutants. The assay has been optimized to use a limited panel of 61 short amplicons (i.e., 33 bp on average) for each of the 16 genes enabling maximum sensitivity for specific cancer detection while increasing the signal-to-noise ratio and minimizing cost that would be associated with longer amplicons reads during downstream sequencing analysis. To further increase the test sensitivity, the authors intentionally partitioned the total amount of purified cfDNA in multiple aliquots, which has been reported to reduce the total amount of DNA per PCR well, while allowing for increasing the mutant molecule fraction compared to normal DNA molecules when considering an initial sample with very low VAF. This strategy allowed for increasing the signal-to-noise ratio and eventually detect lower prevalence mutations. The second component of the assay focuses on evaluating the level of eight different proteins (cancer antigen 125, carcinoembryonic antigen, cancer antigen 19-9, prolactin, hepatocyte growth factor, osteopontin, myeloperoxidase, and tissue inhibitor of metalloproteinases 1) with high potential to discriminate cancer from healthy controls using a single immune assay platform. Together, the mutations data and the protein level are incorporated into a logistic regression algorithm to build the CancerSEEK score and predict cancer types and location when possible. A total of 1005 cancer patients across stages I–III (20% stage I, 49% stage II, and 31% stage III) and 812 healthy individuals were tested using the developed assay which showed remarkable performances at a specificity greater than 99% (with only seven false positives) and an overall sensitivity above 62% (with 379 false negatives), resulting in an AUC of 0.91%. (Overall analytical performances of CancerSEEK are reported in Table 3). CancerSEEK was able to detect diseases across the stage with clinically relevant capabilities showing a sensitivity of 47% for stage I, 63% for stage II, and 70% for stage III. The overall sensitivity of the assay to detect tumour by types was significantly different depending on the organs, which was best for ovary (98.1%) and liver (97.7%) cancers while being the lowest for lung (58.7%) and breast cancers (33.5%). Based on the reported performance (averaged sensitivity of 62.3% at a specificity of 99.14%), the overall PPV of CancerSEEK was estimated to a minimum of 59.4% when considering screening the Australian population in the 55–64 age-restricted group using (Table 8). This value is underestimated and should be over 60% as the prevalence of stomach cancer was not available and could not be included in the calculation. One important aspect of a multi-cancer liquid biopsy detection assay is the ability to identify the tumour of origin when a test returns positive to guide clinicians and allow adequate follow-up. With this regard, CancerSEEK was able to accurately predict the tumour of origin for 63% of the patients that tested positive, with the best prediction for colorectal, pancreatic, and ovary tumours (84%, 81%, and 70%, respectively), and the lowest for lung and liver cancers (39% and 44%, respectively). As opposed to mutations analysis only, the protein level component of the assay plays a major role in locating the organ of origin as genetic mutations in driver genes are usually not tissue-specific—highlighting the importance of the multi-analyte analysis approach. The authors have also estimated the price of such a test to be under 500 US$ being comparable to a standard of care screening methods for a single cancer type, such as colonoscopy. With all these abilities, the CancerSEEK platform holds great opportunities for being implemented as an stMECD test for screening.

## 6.2. Methylation-Based Assay

### 6.2.1. PanSEER

PanSEER is a recent promising blood-based cancer screening test relying on a large panel of ctDNA-methylation biomarkers for the early detection of five major cancer types (colorectal, oesophagal, liver, lung, and stomach), up to four years before conventional diagnostic programs [64]. The assay has been developed to interrogate 477 cancer-specific differentially methylated regions (DMRs, associated with 657 genes and covering 10,613 CpGs sites) at high sequencing depth using semi-targeted PCR libraries constructed from bisulfite-converted cfDNA and analyzed using next-generation sequencing. The assay showed a high detection sensitivity as it was able to capture low cancer DNA fraction down to 0.01%. Combinatorial classifiers then generate prediction scores of cancer-based on the methylation status of the interrogated regions.

One appealing aspect of the PanSEER study for evaluating early cancer detection was the opportunity to retrospectively interrogate blood samples from a large longitudinal cohort including individuals who were initially asymptomatic (i.e., the Taizhou longitudinal study of 123,115 healthy participants) and who later developed a disease that was detected by conventional diagnosis within four years (or remain healthy). The analyzed cohort included 191 of these ‘pre-diagnosis’ patients (including 35 colorectal, 45 oesophagal, 29 liver, 47 lung, and 35 stomach cancers), 414 participants that remained healthy, and an additional set of 223 ‘post diagnosis’ patients who already had cancer (including 7 colorectal, 68 oesophagal, 23 liver, 56 lung and 69 stomach cancers). Half of the samples were randomly assigned to a training set used to generate the cancer classifiers, while the rest were left out for a test set used to independently validate the final developed models. The initial training set was randomly split into two different sub-sets—one for training a cancer classifier and the other for its validation. To prevent potential bias and of using a single training set for fitting the classifier and subsequent misclassification, this process was repeated 1000 times to generate a robust and final model averaging all classifiers’ prediction score output. At a fixed specificity of 95%, this final model achieved a sensitivity of 88.2% in the post-diagnosis group and 91.4% in the pre-diagnosis group using the training set samples. At a fixed specificity of 96%, the analysis of the independent left-aside test set yielded an overall sensitivity of 87.6% (AUC = 0.97) in the post-diagnosis group, and 94.9% (AUC = 0.99) in the pre-diagnosis, together with consistent sensitivity for patients diagnosed with a disease from 1 to 4 years later. Importantly, the authors report similar performances when the model analysed either early and late-stage samples across the entire cohort (overall analytical performances of PANSEER are reported in Table 4). The overall PPV of this assay was estimated to a minimum of 12.1% when considering screening the Australian population in the 55–64 age-restricted group (Table 8). This value is underestimated and should be higher as the prevalence of stomach cancer was not available and could not be included in the calculation. Compared to DEEPGEN and CancerSEEK, PanSEER estimated that PPV is five times lower, which can be reflected in the reduced number of targeted cancer type as well as a weaker specificity.

Altogether, this preliminary study underlines the robust ability of the PanSEER assay to identify individuals with an existing malignant growth across five cancer types but who remained asymptomatic to standard-of-care diagnostic approach. As such, PanSEER clearly shows great potential for an earlier stage of diseases detection in screening settings. However, it has been developed to target a panel of methylation biomarkers that are common signatures of multiple cancers only and is thus not able to predict the organ of origin. This could be achievable by including additional epigenetic markers that are highly tissue-specific and provide high potential for tumour tissue prediction [65]. The low input of initial cfDNA required (i.e., with a reported median amount of 12 ng) along with the limited number of targeted regions allows cost reduction as opposed to other tests interrogating larger panels of markers [66]. PanSEER assay is believed to be implemented as a first-line screening test and guide positive patients to undergo more comprehensive examination such as body imaging for tissue mapping, a process that could be costly. Further independent prospective studies are still needed to fully validate this new methodology.

#### 6.2.2. cfMeDIP-Seq

The ability to increase the analytical sensitivity required to detect trace abundance of ctDNA while limiting potential costs and errors associated with bisulfite sequencing methods can be challenging. To this end, one clever strategy has been implemented by *Shen* et al. [65] where they developed an immunoprecipitation-based assay for specific enrichment of methylated cfDNA followed by high-throughput sequencing analysis (cfMeDIP–seq). Although this protocol does not allow resolution down to single-CpG, it can provide methylation information of larger genomic region (~100bp) without using any DNA bisulfite conversion. The cost of this protocol from cfDNA extraction to sequencing libraries preparation has been estimated to be around $150, which does not include additional sequencing costs [67]. This methodology enables the recovery of very low abundance (>0.001%) of large-scale methylation-ctDNA profiles that are tumour specific using low input of initial DNA (1–10 ng). The initial study was composed of a discovery cohort of 189 plasma samples across seven different cancers (pancreatic, colorectal, breast, lung, renal, bladder cancer, and acute myeloid leukaemia). The authors have first generated cfMeDIP-seq profile from these cfDNA samples to identify differentially methylated regions (DMRs) specific for each cancer type compared to healthy control. Rounds of machine learning analysis were then used to evaluate the ability of such DMRs to classify cancer types. To do so, the cohort was randomly separated into a training (80%) set and a validation set (20%). The training set was used to select the top 300 DMRs for each cancer versus others and to build seven associated cancer-specific classifiers and a healthy-specific one. These models were then used to assign methylation score to the test set samples and to predict their specific tumour type. The performance of the associated classifiers was calculated using AUC receiver operating characteristic curves (AUROC). For optimal classification, this process was repeated 100 times using randomly generated training-test sets producing 100 classifiers for each class. The ensemble was finally applied to predict cancer classes of an independent cohort of 199 samples across acute myeloid leukaemia (AML, *n* = 35), pancreatic cancer (PDAC, *n* = 47), lung cancer (LUC, *n* = 55), and healthy controls (*n* = 62), resulting in high mean AUROC value to classify each cancer versus all-others (0.980 for AML, 0.918 for PDAC, 0.971 for LUC, and 0.969 for healthy samples). Most importantly, the classifiers were yielding similar prediction accuracy when comparing early stage versus late-stage samples for PDAC (0.914 vs. 0.92) and LUC (0.975 vs. 0.966). Together, the cfMeDIP-seq method has been proved to be efficient to retrieve large scale methylation signatures from ctDNA that are highly specific tumour types when carefully selected by machine learning models. (Overall, analytical performances are reported in Table 5).

This whole methodology was validated in a separate study for detecting renal cell carcinoma (RCC) from blood and urine, including early-stage samples [68]. This study included 99 RCC samples (69 from plasma and 30 from urine), 28 non-cancer control (13 from plasma and 15 from urine), with an additional 21 Urothelial bladder cancer (UBC) plasma samples to evaluate the ability of the methods to distinguish between different genitourinary cancers. RCC samples of early stages (I and II) composed a third of the plasma samples and two-thirds of the urine samples. A hundred training-test sets randomly selected were used to generate the classifiers based on the top 300 DMRs, yielding a nearly perfect mean AUROC value to differentiate plasma RCC from control samples (0.99), and plasma RCC from UBC samples (0.979), while the classifier performance was lower for classifying urine RCC from control samples (0.858).

An additional separate study was conducted to evaluate the performance of cfMeDIP-seq method to identify highly specific plasma methylation signatures to detect and discriminate intracranial tumours [69]. Using the previously published cfMeDIP-seq data on seven different extracranial tumours and healthy control [65], plus the cfMeDIP-seq profile of 59 glioma patients (477 samples in total), new classifiers were generated as detailed above with 50 different training-test sets. The models were able to classify brain tumours versus all other classes with very high accuracy (AUC = 0.99). Using an extra 166 samples, they finally demonstrated the utility of the methodology to accurately differentiate cfDNA from six different sub-types of primary brain tumours (AUC values ranged from 0.71 for wild-type glioma to 0.95 for hemangiopericytoma), which are difficult to identify using common imaging techniques.

Such body of evidence clearly demonstrates the indisputable properties of DNA-methylation to be organ type and sub-type specific, and its subsequent power to predict tumour of origin from blood ctDNA when applying appropriate computational models. Although no detailed data have been found regarding the sensitivity of the methods at a specific specificity to detect cancers, the reported AUROC to predict cancer and accurately identify the tumour of origin from early-stage samples provides evidence of the potential of cfMeDIP-seq to be implemented as an MECD.

#### 6.2.3. IvyGene^®^

IvyGene^®^ is another recent technology (commercialized by Laboratory for Advanced Medicine) harnessing the power of methylation signatures for early-stage detection of specific cancer from a blood sample. The platform has also been developed using next-generation sequencing data and bioinformatics to identify specific hypermethylated gene targets and build proprietary methylation-based biomarker panels [70]. The test methodology relies on targeted PCR and NGS and has been used in three different blinded studies to validate individual biomarker panels for the specific detection of liver, breast, and colorectal cancer. The results published in a proceeding detailed very high diagnostic performance to accurately detect each cancer type across all stages (I to IV) [71]. More precisely, the respective specificity and sensitivity of each biomarker panel were 97.5% and 95% to detect liver cancer, 96% and 89% to detect breast cancer, and 100% and 93% to detect colorectal cancer (overall analytical performances are reported in Table 6). One strong aspect of Ivygene^®^ studies is that each patient cohort was designed to include benign tumours in addition to other cancer types and healthy control samples, which is an important factor to better represent a population spectrum and provide a more accurate assessment of how the test will perform in real settings.

#### 6.2.4. GRAIL

In that technological space, the biotech company GRAIL represents a significant shift with their ambitious circulating cell-free genome atlas (CCGA) study, a large-scale population investigation for cancer cfDNA signatures discovery, to develop a blood-based cfDNA multi cancer early detection assay. In a first sub-study, *Liu* et al. [72] conducted a comparative analysis where they showed that the cfDNA methylation approach (i.e., whole-genome bisulfite sequencing, WGBS) outperformed other genomic approaches (i.e., targeted mutation panels and whole-genome sequencing) for cancer detection and organ identification.

In the following CCGA sub-study, these previous WGBS findings and publicly available methylation array data have been used to develop a cfDNA bisulfite sequencing assay targeting a panel of 103,456 informative methylation regions and build classifiers for cancer detection and tissue of origin prediction [73]. The classifiers were trained with 3052 samples (1521 non-cancer and 1531 cancers) and independently validated with a set of 1264 samples (610 non-cancer and 654 cancers) across more than 50 cancer types. The assay specificity was higher than 99% with a reported false-positive rate of 0.7%. The reported sensitivity was 76.4% for a pre-specified set of 12 major cancer types (anus, bladder, colon/rectum, oesophagus, head and neck, liver/bile-duct, lung, lymphoma, ovary, pancreas, plasma cell neoplasm, stomach) and cancer detection sensitivity increased with stages (39% stage-I, 69% stage-II, 75% stage-III, and 92% stage-IV). Importantly, for all the tested samples with a positive cancer signal (*n* = 359), the test was able to predict the tumour of origin with more than 89% accuracy (321/359).

This developed targeted methylation-based assay was further optimized and independently validated in a third and final clinical sub-study using a large cohort of 4077 samples (1254 non-cancers, 2823 cancers) [73]. The reported test performances correlate with the previous sub-study with a specificity of 99.5%, a sensitivity of 76.3% in the pre-specified set of 12 cancers with corresponding sensitivity per stage of 37.1% for stage I, 69.7% for stage II, 86.6% for stage III, and 92.8% for stage IV (no plasma cell neoplasm sample reported for stage IV). The overall tissue of origin prediction accuracy in true positive was 88.7% (overall analytical performances are reported in Table 7). The overall PPV of GRAIL has been estimated to a minimum of 84.2% (Table 8). This value is underestimated and should be higher as the prevalence of multiple cancers targeted by the assay were not available (stomach, plasma cell neoplasm, multiple primaries, urothelial track, gallbladder, sarcoma, other, lymphoid leukemia, myeloid neoplasm, kidney, and thyroid) and could not be included in the calculation.

Compared to the other stMCED platforms, GRAIL is certainly the most powerful and which has been thoroughly validated by large cohorts of participants. Even though the overall sensitivity is similar or lower than the other techniques discussed herein (in particular for early disease stages), the ability to target that many cancer types simultaneously with a false positive rate of only 0.7% would yield the highest PPV compared to other methods targeting fewer diseases with reduced specificity. Indeed, a screening test would significantly benefit from a higher aggregated prevalence—by increasing the number of targeted organs—with moderate sensitivity, rather than favouring sensitivity with a reduced number of interrogated cancers and specificity. The additional ability to predict the tumour of origin with good accuracy is certainly of great additional value for patient health and finances. However, that package of benefits is only possible at an elevated price because a very high number of genomic regions need to be interrogated through sequencing technology, which may be a limit for the uptake of the test by the targeted population.

#### 6.2.5. Methylscape

Despite being highly robust and powerful, all the methodologies reported above entirely rely on PCR and sequencing technologies, involving relatively expensive equipment and well-trained operators, thereby limiting their access in resource-poor countries and remote areas. Knowing that disease screening is only feasible with technologies that could be implemented and sustained in low-resource settings, simpler and cheaper screening methods are still needed for cancer diagnostic intervention beyond the boundaries of the first world [2]. In that technological space, one emerging methodology is Methylscape—a novel type of universal cancer biomarkers characterized by a specific Methylation landscape across the genome [74,75]. They reported that the global epigenetic reprogramming arising during malignant transformation (i.e., DNA hyper- and hypo-methylation)—characteristic of most cancer types—was affecting the physicochemical properties of DNA and significantly increasing its gold physisorption. By taking advantage of that phenomenon, a simple interfacial biosensing assay has been developed to electrochemically quantify direct DNA absorption level at a bare gold electrode surface to differentiate normal from cancer epigenomes. In a pilot study using only 5 pg of plasma-derived cfDNA, the assay was able to differentiate healthy controls (*n* = 45) from advanced colorectal and breast cancer (*n* = 100) samples with relatively good performance (AUROC = 0.887). However, further development is still required to assess the potential of Methylscape to detect earlier stages of diseases and independent validations including a larger panel of cancer types across multiple cancer stages are still needed to be done.

Although Methylscape showed lower cancer detection accuracy than the other sequencing-based assay described above, its simple methodology enables reduction of sample preparation process to the bare minimum (i.e., label-free, biofunctionalization-free sensor, no bisulfite DNA treatment, no DNA amplification nor sequencing) allowing for putting forward a very simple and yet efficient technology at a significantly reduced cost. Thus, Methylscape could be implemented using portable equipment and could find great opportunities in low-resource settings as a “quick and cheap” first-line screening technology for cancer detection.

## 7. Clinical Translation of stMCEDs: Summary and Future Perspectives

Conventional multimodal screening programs are mainly limited by the low number of cancers recommended for screening as well as the high cumulative false-positive rate and associated harms resulting from repeated testing. The possibility to use minimally invasive blood tests to screen asymptomatic individuals at-risk for multiple cancer types simultaneously would benefit from a fixed specificity and the aggregated diseases prevalence, both parameters increasing the positive predictive value of the test and its efficiency. As such, stMCED technologies are now emerging as a promising paradigm for increasing earlier disease detection rate with better harm–benefit trade-off and potentially improving population health. However, the development of these technologies must show a positive clinical cost–benefit to allow potential implementation into current screening programs. With this regard, the risk–benefit analysis of these tests should be systematically performed based on diagnostic performances to evaluate their net clinical impact on the healthcare system and patient’s health, management, and finances. For successful translation, the net benefit of any of these tests should outweigh the total risks associated with its use. For instance, a hypothetic low-cost test sensitive enough to detect localized diseases and potentially improve patient survival outcome, but showing a relatively high false-positive rate and/or the inability to identify the tumour of origin would probably remain too risky for screening settings. As such, the burdensome consequences resulting from inaccurate testing would still outweigh the potential health benefit.

While the legitimate clinical utility of DNA-methylation compared to genetic alteration for accurate cancer diagnostic has been debated in the scientific community, there is now unprecedented evidence tipping the balance in favour of epigenetic biomarkers. One important and underappreciated aspect of cfDNA-based liquid biopsy testing is the relationship between the reported sensitivity of these tests and the amount of input DNA required to achieve it. For example, one complete copy of the human genome is equivalent to approximately 3 pg of DNA [77], and an input of 12 ng of would be equivalent to 4000 copies of the genome. Extending this example, a cfDNA sample composed of 20% tumour DNA, where 10% of the tumour DNA has a somatic mutation equals 120 copies of the mutant allele are theoretically available, but only at a single locus. In contrast, as DNA methylation changes are generally a genome-wide phenomenon, the same 12 ng cfDNA containing 20% tumour material would have 800 copies of DNA at multiple loci, enabling a much greater sensitivity, highlighting the power of DNA methylation as a clinical diagnostic. As outlined in the present review, one of the most compelling arguments in favour of DNA methylation is certainly the possibility to identify ctDNA epigenetic signatures that accurately reflect specific organ of origin—clinical information highly beneficial to allow translation of a multi-cancers screening test into current guidelines.

Nonetheless, to correctly infer the clinical cost–benefit of a test, its performances must be thoroughly validated by independent clinical studies. Designing robust studies that faithfully reflect the targeted population can be challenging when investigating early stage of malignancies as diseases prevalence are low and a very large number of samples should be included for statistical significance. In that line, case-control studies often fail to accurately represent the broad spectrum of individuals within risk populations as they usually include retrospective samples from either cancerous or non-cancerous patients and often omit to include other cancer-like patients such as benign neoplasms or inflammatory associated samples. This could raise concern as DNA methylation mechanisms have been reported to also be triggered during such cancer-like responses which could lead to false assessment of a diagnostic performance [78].

Altogether, the overall capabilities of these stMCED technologies are strongly dependent on the selected biomarker panels and richness of patients’ cohorts used for generating the input data to train the core machine learning classifiers. The diagnostic power of these computational methodologies is believed to significantly improve with growing datasets and could potentially achieve optimal performance by combining genetic and epigenetic biomarker panels to eventually satisfy the clinical cost–benefit required for successful implementation. Although these stMCED methodologies are reporting promising diagnostic performances, their overall sensitivities to detect early stage of cancers (i.e., stage I and II) are still underperforming compared to later stage detection. Research in the field should endeavour towards the development of new panels or technologies of early-stage biomarkers—with the capabilities to determine whether a neoplasm tissue will transform into an aggressive tumour or remain benign—to effectively increase earlier detection of malignant diseases and facilitate improved treatment opportunities and clinical outcome of patients.

## 8. Methodology

### 8.1. Literature Search

The cancer diagnostic tests reviewed in this article have been selected from the literature after a digital search on Google Scholar for the following criteria: (1) published online within the last four years (from 2018 onwards), (2) using circulating tumour DNA analysis from liquid biopsy testing, (3) focusing on the detection of multiple cancer types, (4) reporting on the ability to detect early stage of malignant diseases, and (5) using DNA methylation biomarkers for cancer detection. In addition, two distinct methodologies not utilizing DNA methylation biomarkers, but somatic mutation analysis, have also been included in this review to better contrast the performance of diagnostic tests in this space as we believe this information is of value to readers.

### 8.2. Positive Predictive Value (PPV) Analysis

In-silico modelling of a diagnostic test PPV in relation to different sensitivity, specificity, and disease prevalence parameters was performed using the following equation reported by *S. Tenny and M.R. Hoffman* [35]:

$$PPV=\frac{\left(prevalence*sensitivity\right)}{\left(prevalence*sensitivity\right)+\left[\left(1-prevalence\right)*\left(1-specificity\right)\right]},$$

and values were plotted in a series of heatmap graphs using GraphPad Prism 8 (Figure 2).

When possible, the PPV associated with the methodologies discussed in this review was evaluated using Equation (1), and data are shown in Table 8. The calculation was considered feasible for the methodologies reporting on a fixed specificity value for screening multiple cancer types and the associated performance in terms of its sensitivity. Therefore, the PPV was only evaluated for DEEPGEN™, CancerSEEK, PanSEER and GRAIL, while IvyGene^®^, cfMeDIP-sequ, and Methylscape were excluded. The disease prevalence data used to estimate the PPV have been obtained from Cancer Australia’s National Cancer Control Indicators (NCCI) website [76] and are expressed as the 5-year limited duration prevalence between 2010 and 2014. That is, the number of individuals diagnosed with cancer in Australia between 1 January 2010 and 31 December 2014 period and who were still alive at the end of 2014. The data have been further restricted to the age group 55–64 years to represent a healthy population likely to undergo cancer screening programs. The 5-year prevalence data were only available for a limited number of cancer types (i.e., bladder, brain, breast, primary unknown, cervical, colorectal, head and neck, liver, lung, melanoma, non-Hodgkin’s lymphoma, oesophageal, ovarian, pancreatic, prostate and uterine cancers), and data for other cancers (e.g., stomach) were not specified. As such, the PPV could not be estimated to its fullest for methodologies targeting these additional cancers (i.e., CancerSEEK, PanSEER, and mostly GRAIL). The overall 5-year prevalence has been computed separately for each methodology as the sum of the 5-year prevalence of each cancer type a methodology is targeting (only possible when the prevalence data were provided).

## Figures and Tables

**Table 1:** Overview of reported methodologies with an upcoming potential for single test-based multi-cancer early detection. The vertical double line separates methodologies using methylation-based (right) from non-methylation based (left) biomarkers. DMRs: differentially methylated regions, TOO: tissue of origin prediction, AUROC: area under the receiving operating curve, LOD: limit of detection -: not specified or not applicable. Methylscape was not included due to limited early-stage data.

	DEEPGEN™	CancerSEEK	PanSEEER	cfMeDIP-seq	GRAIL	IvyGene^®^
**Biomarker type**	Genomic variants	Genomic variant (1933 mutations, 16 genes) & 8 proteins	477 DMRs (657 genes, 10,613 CpGs)	Enriched DMRs	>100,000 DMRs (1,166,720 CpGs, cover 17.2 Mb)	Targeted panels of methylation biomarkers
**Targeted cancer types**	7	8	5	9 (across 3 sperate studies)	12 pre-specified (>50 sub-types)	3
**Specificity (%)**	95	99.14	96.1	-	99.52	96–100
**Sensitivity (%):** overall stage 1 stage 2 stage 3 stage 4	57 51 58 62 67	62.3 48 63 70 -	~95 ~95 (s1-2) ~95 (s3-4)	- - - - -	51.5 16.8 40.4 77 90.1	89–95 - - - -
**AUROC:** overall stage 1 stage 2 stage 3 stage 4	0.9 0.88 0.9 0.92 0.94	0.91 - - - -	~0.99 ~0.99 (s1-2) ~0.99 (s3-4)	0.91 to 0.99 (s1-2) 0.92 to 0.99 (s3-4)	-	-
**TOO capacity** (depends on organs)	No	Yes (median 63%)	No	Only	Yes (overall 88.7%)	Only
**cfDNA input (ng)**	-	-	~12	1–10	-	-
**LOD (% ctDNA)**	> 0.09	-	>0.01	>0.001	-	-

**Table 2:** Detailed analytical performance of the DEEPGEN™ assay [61]. AUROC: area under the receiving operating curve.

DEEPGEN™	Cohort Size (Healthy = 415)	Specificity (%)	Sensitivity (%)	AUROC
**All cancer**				
stage 1	70	95	51	0.88
stage 2	55	95	58	0.9
stage 3	73	95	62	0.92
stage 4	27	95	67	0.94
Overall	260	95/99	57/43	0.9
**Bladder**				
Overall	25	95/99	80/32	
**Prostate**				
Overall	29	95/99	72/62	
**Lung**				
Overall	30	95/99	67/53	
**Liver**				
Overall	27	95/99	63/41	
**Pancreatic**				
Overall	40	95/99	52/38	
**Colorectal**				
Overall	66	95/99	42/27	
**Breast**				
Overall	43	95/99	30/16	

**Table 3:** Detailed analytical performance of the CancerSEEK assay [63]. TOO: tissue of origin, AUROC: area under the receiving operating curve.

CancerSEEK	Cohort Size (Healthy = 812)	Specificity (%)	Sensitivity (%)	AUROC	TOO Prediction
**All cancer**		99.14			
stage 1	199		48		
stage 2	497		63		
stage 3	309		70		
Overall	1005		62.3	0.91	63%
**Ovary cancer**					
stage 1	9		88.9		
stage 2	4		100.0		
stage 3	41		100.0		
Overall	54		98.1		79%
**Esophagus cancer**					
stage 1	5		20.0		
stage 2	29		86.2		
stage 3	11		45.5		
Overall	45		68.9		46% (with stomach)
**Lung cancer**					
stage 1	46		43.5		
stage 2	27		66.7		
stage 3	31		74.2		
Overall	104		58.7		39%
**Liver cancer**					
stage 1	5		100.0		
stage 2	19		100.0		
stage 3	20		95.0		
Overall	44		97.7		44%
**Pancreatic cancer**					
stage 1	4		25.0		
stage 2	83		73.5		
stage 3	6		83.3		
Overall	93		72.0		81%
**Colorectal cancer**					
stage 1	77		42.9		
stage 2	191		72.3		
stage 3	120		67.5		
Overall	388		64.9		84%
**Breast cancer**					
stage 1	32		37.5		
stage 2	114		25.4		
stage 3	63		46.0		
Overall	209		33.5		63%
**Stomach cancer**					
stage 1	21		71.4		
stage 2	30		66.7		
stage 3	17		82.4		
Overall	68		72.1		46% (with oesophagus)

**Table 4:** Detailed analytical performance of the PanSEER assay [64]. AUROC: area under the receiving operating curve.

PanSEER	Cohort Size (Healthy = 207)	Sample Number Per Stage: (1–2)–(3–4)	Specificity (%)	Sensitivity (%)	AUROC
**All cancer**			96.10		
Post diagnosis	113	32–80		87.6	0.97
Pre diagnosis:	98			94.9	0.99
0–1 year before	21	5–13		95.2	0.99
1–2 year before	23	6–17		95.7	0.99
2–3 years before	31	10–17		93.6	0.99
3–4 years before	23	8–9		95.7	0.99
**Esophagus**					
stage 1–2	46				
stage 3–4	63				
Overall	113				
**Lung**					
stage 1–2	18				
stage 3–4	80				
Overall	103				
**Liver**					
stage 1–2	7				
stage 3–4	43				
Overall	52				
**Colorectal**					
stage 1–2	21				
stage 3–4	16				
Overall	42				
**Stomach**					
stage 1–2	44				
stage 3–4	54				
Overall	104				

**Table 5:** Detailed analytical performance of the cfMeDIP-seq assay (reported from three separate studies delimited by the double lines) [65,68,69]. TOO: tissue of origin, AUROC: area under the receiving operating curve.

cfMeDIP-seq	Cohort Size in Sets: (Train/Test)—Validation	Accuracy to Predict Cancer with TOO (AUROC)
**Lung cancer**		
stage 1–2	32	0.975
stage 3–4	(22)–23	0.966
Overall	(25)–55	0.971
**Pancreatic cancer**		
stage 1–2	(23)–15	0.914
stage 3–4	(1)–32	0.92
Overall	(24)–47	0.918
**Acute myeloid leukaemia**		
Overall	35	0.98
**Healthy**		
Overall	(24)–62	0.969
**Colorectal cancer**		
stage 1–2	(1)	-
stage 3–4	(21)	-
Overall	(23)	-
**Bladder cancer**		
Overall	(20)	-
**Renal cancer**		
Overall	(20)	-
**Renal cancer**		
stage 1–2	(33)	-
stage 3–4	(66)	-
Overall	(99)	0.99
**Intracranial Glioma**		
Overall	(59)	0.99

**Table 6:** Detailed analytical performance of the IvyGene^®^ technology for the detection of liver, breast, and colorectal cancers [71]. TOO: tissue of origin.

IvyGene^®^ (Laboratory for Advanced Medicine)	Cohort Size	Specificity (%)	Sensitivity (%): Predict Cancer & TOO Accuracy
**Liver cancer**			
Overall (stage 1–4)	60	97.5	95
**Healthy (control)**			
Overall	30		
**Benign liver (control)**			
Overall	10		
**Other cancers (control)**			
Overall	30		
**Breast cancer**			
Overall (stage I-IV)	65	96	89
**Healthy (control)**			
Overall	39	95	
**Benign breast (control)**			
Overall	15	100	
**Other cancers (control)**			
colorectal	11		
liver	9		
lung	12		
Overall	32	96	
**Colorectal cancer**			
Overall (stage 1–4)	68	100	93 (67–100)
**Healthy (control)**			
Overall	42		
**Benign colorectal (control)**			
Overall	14		
**Other cancers (control)**			
breast	10	100	
liver	10	100	
lung	10	100	
Overall	30		

**Table 7:** Detailed analytical performance of GRAIL technology [73]. The data from the 12 pre-specified cancers are listed first and separated from the remaining cancer types by a double line. The remaining cancer types are listed in decreasing order according to their respective overall sensitivity value. TOO: tissue of origin.

GRAIL	Cohort Size (Healthy = 1254)	Specificity (%)	Sensitivity (%)	TOO Prediction Accuracy (%) (For True Positive)
**All cancer**		99.52		
Stage 1	849		16.8	
Stage 2	703		40.4	
Stage 3	566		77.0	
Stage 4	618		90.1	
Overall	2823		51.5	88.7
**Liver/bile-duct**				
Stage 1	6		100.0	
Stage 2	10		70.0	
Stage 3	9		100.0	
Stage 4	20		100.0	
Overall	46		93.5	93.0
**Head & neck**				
Stage 1	19		63.2	
Stage 2	17		82.4	
Stage 3	19		84.2	
Stage 4	50		96.0	
Overall	105		85.7	93.3
**Esophagus**				
Stage 1	8		12.5	
Stage 2	17		64.7	
Stage 3	34		94.1	
Stage 4	40		100.0	
Overall	100		85.0	-
**Pancreatic**				
Stage 1	21		61.9	
Stage 2	20		60.0	
Stage 3	21		85.7	
Stage 4	73		95.9	
Overall	135		83.7	-
**Ovary**				
Stage 1	10		50.0	
Stage 2	5		80.0	
Stage 3	31		87.1	
Stage 4	19		94.7	
Overall	65		83.1	70.4
**Colorectal**				
Stage 1	30		43.3	
Stage 2	40		85.0	
Stage 3	66		87.9	
Stage 4	64		95.3	
Overall	206		82.0	98.8
**Anus**				
Stage 1	4		25.0	
Stage 2	4		75.0	
Stage 3	13		100.0	
Stage 4	1		100.0	
Overall	22		81.8	77.8
**Lung**				
Stage 1	96		21.9	
Stage 2	44		79.5	
Stage 3	118		90.7	
Stage 4	145		95.2	
Overall	404		74.8	91.7
**Plasma cell neoplasm**				
Stage 1	17		64.7	
Stage 2	16		87.5	
Stage 3	14		64.3	
Stage 4	-		-	
Overall	47		72.3	-
**Stomach**				
Stage 1	6		16.7	
Stage 2	6		50.0	
Stage 3	5		80.0	
Stage 4	12		100.0	
Overall	30		66.7	-
**Lymphoma**				
Stage 1	33		27.3	
Stage 2	48		58.3	
Stage 3	46		71.7	
Stage 4	46		60.9	
Overall	174		56.3	-
**Bladder**				
Stage 1	6		33.3	
Stage 2	11		9.1	
Stage 3	4		75.0	
Stage 4	2		100.0	
Overall	23		34.8	87.5
**Unknown primary**				
Stage 1	-		-	
Stage 2	1		100.0	
Stage 3	2		50.0	
Stage 4	13		100.0	
Overall	18		94.4	-
**Multiple primaries**				
Stage 1	2		100.0	
Stage 2	5		60.0	
Stage 3	6		100.0	
Stage 4	6		83.3	
Overall	19		84.2	-
**Urothelial track**				
Stage 1	2		0.0	
Stage 2	-		-	
Stage 3	-		-	
Stage 4	8		100.0	
Overall	10		80.0	-
**Cervix**				
Stage 1	12		58.3	
Stage 2	5		100.0	
Stage 3	7		100.0	
Stage 4	1		100.0	
Overall	25		80.0	35.0
**Gallbladder**				
Stage 1	2		0.0	
Stage 2	3		33.3	
Stage 3	4		75.0	
Stage 4	8		100.0	
Overall	17		70.6	-
**Sarcoma**				
Stage 1	10		40.0	
Stage 2	2		100.0	
Stage 3	10		50.0	
Stage 4	7		85.7	
Overall	30		60.0	-
**Other**				
Stage 1	11		18.2	
Stage 2	3		100.0	
Stage 3	18		72.7	
Stage 4	18		61.1	
Overall	59		50.8	-
**Melanoma**				
Stage 1	2		0.0	
Stage 2	2		0.0	
Stage 3	3		0.0	
Stage 4	6		100.0	
Overall	13		46.2	100.0
**Lymphoid leukemia**				
Stage 1	-		-	
Stage 2	-		-	
Stage 3	-		-	
Stage 4	-		-	
Overall	51		41.2	-
**Breast**				
Stage 1	265		2.6	
Stage 2	181		47.5	
Stage 3	55		85.5	
Stage 4	22		90.9	
Overall	524		30.5	96.9
**Uterus**				
Stage 1	120		16.7	
Stage 2	10		30.0	
Stage 3	23		73.9	
Stage 4	4		100.0	
Overall	157		28.0	-
**Myeloid neoplasm**				
Stage 1	-		-	
Stage 2	-		-	
Stage 3	-		-	
Stage 4	-		-	
Overall	10		20.0	-
**Kidney**				
Stage 1	61		4.9	
Stage 2	9		22.2	
Stage 3	7		14.3	
Stage 4	22		54.5	
Overall	99		18.2	77.78
**Prostate**				
Stage 1	95		3.2	
Stage 2	243		4.9	
Stage 3	50		14.0	
Stage 4	30		83.3	
Overall	420		11.2	-
**Thyroid**				
Stage 1	11		0.0	
Stage 2	1		0.0	
Stage 3	1		0.0	
Stage 4	1		0.0	
Overall	14		0.0	-

**Table 8:** Estimated positive predictive value (PPV) for four reviewed multi-cancer early detection tests (DEEPGEN™, CancerSEEK, PanSEER, and GRAIL) in a function of the reported test performances and the 5-year limited duration prevalence of cancers in the Australian population between 2010 and 2014 (restricted to the age group 55–64 years). The overall 5-year prevalence rate (*) is different for each assay and has been computed as the sum of the 5-year prevalence rate of each cancer type a test is targeting (only including the prevalence data provided in [76]). The corresponding values are listed in the table below each test name.

Epidemiologic Data for the Australian Population (2010–2014)—Restricted to the Aged Group 55–64 Years [76]		DEEPGEN™ at 95%/99% Specificity * 3604 (*n*/100,000)		CancerSEEK ^a^ at 99.14% Specificity * 1981 (*n*/100,000)		PanSEER ^a^ at 96.1% Specificity * 560 (*n*/100,000)		GRAIL ^b^ at 99.52% Specificity * 4716.1 (*n*/100,000)	
Cancer Type	5-Year Prevalence Rate (*n*/100,000)	Sensitivity (%)	PPV (%)	Sensitivity (%)	PPV (%)	Sensitivity ^c^ (%)	PPV (%)	Sensitivity (%)	PPV (%)
Overall	* Specific value for each assay	57.0/43.0	29.9/61.7	62.3	>59.4	94.9	>12.1	51.5	>84.2
Bladder	40.3	32.0/80.0	0.6/1.3	-	-	-	-	34.8	2.8
Brain	23.0	-	-	-	-	-	-	-	-
Breast (female only)	1319.4	16.0/30.0	7.4/17.6	33.5	34.3	-	-	30.5	45.9
Primary unknown	22.3	-	-	-	-	-	-	99.4	4.4
Cervical	0.0	-	-	-	-	-	-	80.0	5.4
Colorectal	367.9	42.0/27.0	3.0/9.1	64.9	21.8	n.s	-	82.0	38.7
Head and neck	163.3		-	-	-	-	-	85.7	22.6
Liver	38.7	63.0/41.0	0.5/1.6	98.7	4.3	n.s	-	93.5	7.0
Lung	132.9	67.0/53.0	1.8/6.6	58.7	8.3	n.s	-	74.8	17.2
Melanoma	419.8	-	-	-	-	-	-	46.2	28.9
Non-Hodgkin lymphoma	145.3	-	-	-	-	-	-	56.3	14.6
Oesophageal	20.5	-	-	68.1	1.6	n.s	-	85.0	3.5
Ovarian (female only)	73.2	-	-	98.1	7.7	-	-	83.1	11.3
Pancreatic	28.4	52.0/38.0	0.3/1.1	72	2.3	-	-	83.7	4.7
Prostate (male only)	1676.4	72.0/62.0	19.7/51.4	-	-	-	-	11.2	28.5
Uterine (female only)	233.3	-	-	-	-	-	-	28.0	12.0

**a**. Stomach cancer was not included as the 5-year prevalence data was not reported in [76]. **b**. Stomach, plasma cell neoplasm, multiple primaries, urothelial track, gallbladder, sarcoma, other, lymphoid leukemia, myeloid neoplasm, kidney, and thyroid were not included as the 5-year prevalence data were not reported in [76]. **c**. Sensitivity value reported in the context of pre-diagnosis data.

## Data Availability

The majority of the data were collected from publicly available papers as listed in the references to create the tables reported in this review article. The positive predictive value (PPV) calculation were derived from publicly available statistics (see methodology Section 8) and the corresponding excel files can be requested by contacting the corresponding authors.
